# PCR sensitivity for *Mycoplasma pneumoniae* detection in nasopharyngeal and oropharyngeal swabs: a comparative study

**DOI:** 10.1128/jcm.00458-25

**Published:** 2025-07-03

**Authors:** Daisuke Kitagawa, Shin Nishihara, Masayuki Murata, Mai Onishi, Takahiro Mori, Soshi Hachisuka, Tenshin Okubo, Naohiro Yamamoto, Hiroki Nishikawa, Masayuki Onaka, Rika Suzuki, Soma Suzuki, Ayu Yamamoto, Ritsuki Uejima, Fumihiko Nakamura, Sayaka Yoshida, Taito Kitano

**Affiliations:** 1 Department of Laboratory Medicine, Nara Prefecture General Medical Center 36927, Nara, Japan; 2 Sukusuku Kid’s Clinic, Nara, Japan; 3 Department of Pediatrics, Nara Prefecture General Medical Center 36927, Nara, Japan; National Institute of Allergy and Infectious Diseases, Bethesda, Maryland, USA

**Keywords:** *Mycoplasma pneumoniae*, diagnosis, sensitivity and specificity, rapid diagnostic tests, polymerase chain reaction

## Abstract

The differential impact of sample type on polymerase chain reaction (PCR) detection of *Mycoplasma pneumoniae* (MP) has rarely been investigated. The study aimed to evaluate the diagnostic performance of PCR for the detection of MP and to measure MP DNA load between nasopharyngeal and oropharyngeal swabs. Nasopharyngeal and oropharyngeal samples were obtained simultaneously to evaluate their diagnostic performance in children with suspected MP. Two commercially available PCR tests, multiplex PCR and Smart Gene Myco, were used to analyze the nasopharyngeal and oropharyngeal samples, respectively. Furthermore, real-time PCR (RT-PCR) tests were conducted on both sample residues to validate the results. In total, 422 participants underwent simultaneous PCR testing using nasopharyngeal and oropharyngeal swabs; 139 samples (32.9%) from nasopharyngeal swabs and 176 samples (41.7%) from oropharyngeal samples that tested positive using commercially available tests. RT-PCR tests were positive for 136 (32.2%) nasopharyngeal and 183 (43.4%) oropharyngeal residual samples. With the RT-PCR test of the residual extract from oropharyngeal swabs as a reference, the sensitivity and specificity of detecting MP were 74.9% (95% confidence interval 67.9%–81.0%) and 99.2% (97.0%–99.9%) with the multiplex PCR test on nasopharyngeal swabs, and 96.2% (92.3%–98.4%) and 100.0% (98.5%–100.0%) with the Smart Gene Myco on oropharyngeal samples. A negative correlation was observed between fluoroquinolone use and oropharyngeal DNA loads (*P* = 0.004). The sensitivity of MP detection was significantly better in oropharyngeal samples than in nasopharyngeal samples. This study indicates that oropharyngeal samples should be used to detect MP rather than nasopharyngeal samples.

**IMPORTANCE:**

Obtaining the best sample is crucial for the accurate diagnosis of *Mycoplasma pneumoniae* (MP) and timely and appropriate treatment. This study aimed to assess the diagnostic performance of MP detection using polymerase chain reaction (PCR) tests between nasopharyngeal and oropharyngeal samples. This study showed that the sensitivity of detecting MP was 74.9% (95% confidence interval 67.9%–81.0%) with a commercially available PCR test on nasopharyngeal swabs, and 96.2% (92.3%–98.4%) with a commercially available PCR test on oropharyngeal samples. The sensitivity of MP detection was significantly better in oropharyngeal samples than in nasopharyngeal samples. This study supports the idea that oropharyngeal samples should be used to detect MP. The results contribute to guidance in the recommendation regarding sampling methods to detect MP. Accurate identification of MP is crucial not only for timely and appropriate antimicrobial treatment but also for efficient epidemiological surveillance.

## INTRODUCTION

*Mycoplasma pneumoniae* (MP) is a common cause of lower respiratory tract infections (LRTI) in school-aged children and adolescents (1, 2). The mainstay of treatment for MP-induced pneumonia is non-beta-lactam antimicrobial agents. Given the overlapping symptoms and clinical presentations of LRTI caused by MP with those of respiratory viruses and bacteria (e.g., *Streptococcus pneumoniae* and *Haemophilus influenzae*) (3), each needing different antimicrobial treatments (i.e., no antimicrobials for viral LRTI and beta-lactam for bacterial pneumonia), a rapid and accurate diagnosis of causal pathogens is critical.

Although obtaining the best sample is crucial for the accurate diagnosis of MP and timely and appropriate treatment, the effect of sample type on polymerase chain reaction (PCR) detection of MP has rarely been investigated (4–6). Given the relative lack of evidence regarding optimal sampling type for PCR detection of MP, the United States Centers for Disease Control and Prevention and other guidelines accept both nasopharyngeal and oropharyngeal samples for MP detection (7, 8). However, to our knowledge, the largest comparative study conducted to date on PCR detection of MP using nasopharyngeal and oropharyngeal swabs included only 58 MP-positive patients (6). Moreover, the DNA load was not evaluated in this study. Consequently, data with a larger sample size and bacterial quantification could provide a more detailed evaluation of the factors responsible for the discrepancy in detection using different sampling methods. To establish evidence regarding the optimal sample to detect MP, further studies with a larger sample size, along with measurement of DNA load, are needed.

Diagnostic performance is not only affected by the sampling method but also by other factors, including prior antimicrobial treatment and antimicrobial resistance patterns (9). Macrolide-resistant MP is an emerging global issue (10). Evaluation of the impact of these factors on diagnostic performance and DNA load could help develop an optimal diagnostic strategy for MP. The primary objective of our study was to evaluate the diagnostic performance of PCR detection of MP as well as the DNA loads of MP between nasopharyngeal and oropharyngeal samples. The secondary objective was to assess the impact of prior antimicrobial therapy as well as the impact of the presence of macrolide-resistance gene mutations on bacterial load.

## MATERIALS AND METHODS

### Study design and settings

This study was conducted between July 25 and December 27, 2024, in Nara, Japan. Two pediatric facilities were included (a pediatric clinic and a pediatric department in a referral hospital). The eligibility criteria were (i) patients ≤ 18 years old with fever and/or respiratory symptoms and (ii) those who simultaneously underwent nasopharyngeal and oropharyngeal PCR tests for MP at a study center. Before and during the study period, both study centers conducted multiplex PCR tests using nasopharyngeal swabs from symptomatic patients to detect respiratory pathogens as part of standard local practice. The SpotFire respiratory panel (bioMérieux, Marcy-l'Étoile, France) was used in the clinic, and FilmArray respiratory panel 2.1 (bioMérieux) was used in the referral hospital to facilitate appropriate cohorting. The multiplex PCR test detected multiple respiratory pathogens, including MP, using nasopharyngeal swabs and universal transport medium (UTM) Nasopharyngeal Sample Collection Kit (Copan, Brescia, Italy). The limit of detection (LOD) for MP was 2.1 copies/µL for the SpotFire respiratory panel, 0.46 copies/µL for the FilmArray respiratory panel 2.1. In response to the epidemic surge of MP in 2024 and the high prevalence of macrolide-resistant MP in Japan (11, 12), the two study centers added the Smart Gene Myco (MIZUHO MEDY Co., Ltd., Tosu, Japan), which used oropharyngeal swabs (Nipro sponge swab TYPE L, Nipro, Osaka, Japan) and extraction buffer solution containing surfactants and chaotropic salts (MIZUHO MEDY Co., Ltd.), to simultaneously analyze nasopharyngeal swabs from children with respiratory symptoms and clinically suspected MP as a standard local practice. The Smart Gene Myco is a point-of-care test, which enables rapid detection of the nucleic acid of MP and macrolide-resistance gene mutations (positions 2063 and 2064 in domain V of the 23S ribosomal RNA gene) from oropharyngeal swabs using QProbe PCR (13). The LOD for MP was 10 copies/µL. The Smart Gene Myco was introduced to improve the sensitivity of MP detection amid the local MP epidemic and support timely and appropriate antimicrobial prescriptions guided by macrolide-resistance gene mutation results. Sample collection and testing were performed according to relevant regulatory guidelines (14–16). Physicians trained in pediatric sample collection (either board-certified pediatricians or resident physicians) collected the pediatric samples. Both nasopharyngeal and oropharyngeal samples from each individual were collected by the same physician. These commercially available PCR tests from a nasopharyngeal and an oropharyngeal sample in a single individual were run in parallel. Both nasopharyngeal and oropharyngeal samples were frozen within a day after examination (17). The samples were stored at −80°C in the hospital and −18°C in the clinic. All paired nasopharyngeal and oropharyngeal samples were stored and transported under the same condition.

Residual UTM from the nasopharyngeal swab and residual extract from the oropharyngeal swab were sent to a laboratory at MIZUHO MEDY Co., Ltd., Tosu, Japan with a freezer box (no thawing was observed in any samples). DNA was extracted using the QIAamp DNA Mini Kit (Qiagen, Hilden, Germany). In-house real-time PCR (RT-PCR) tests were conducted using the AriaMx Real-Time PCR System (Agilent Technologies Inc., Carpinteria, CA, USA) for all UTM from nasopharyngeal samples and residual extracts from oropharyngeal samples to validate MP detection results from the multiplex PCR tests (using nasopharyngeal samples). DNA loads (copies/μL) were also determined. The RT-PCR assay targeted the CARDS toxin gene. The primers and probe used were forward primer 5′-TTTGGTAGCTGGTTACGGGAAT-3′, reverse primer 5′-GGTCGGCACGAATTTCATATAAG3′, and probe 5′-FAM- TGTACCAGAGCACCCCAGAAGGGCT-BHQ1-3′. The assay was developed and validated in-house, based on previously published methods (18). For quantification, synthetic DNA standards containing the target sequence were used to generate a standard curve from 10^6^ to 10^1^ copies/test. Each dilution was tested in duplicate to ensure reproducibility. Quality control measures included positive controls using MP reference strain M129 (ATCC 29342) and negative extraction controls in each run. The assay demonstrated linearity across the measured range (*R*^2^ >0.99). No cross-reactivity with other respiratory pathogens was observed during internal validation using clinical and reference strains.

### Statistical analysis

The RT-PCR test of residual extracts from oropharyngeal swabs was used as a reference to evaluate the sensitivity and specificity of detecting MP in each test: (i) a multiplex PCR test (SpotFire respiratory panel or FilmArray respiratory panel) using nasopharyngeal swabs, (ii) Smart Gene Myco using oropharyngeal samples, and (iii) RT-PCR test of residual UTM from nasopharyngeal samples were calculated with 95% confidence intervals (CI). McNemar’s tests were conducted to compare the sensitivities of two different testing strategies (19).

The DNA loads between the nasopharyngeal and oropharyngeal swabs were compared using the Wilcoxon signed-rank test, and the correlation was evaluated using Spearman’s rank correlation. To assess the impact of prior antimicrobial treatment and the detection of macrolide resistance gene mutations on oropharyngeal and oropharyngeal DNA loads using RT-PCR tests, generalized linear models were constructed with explanatory variables including age group, sex, presence of comorbidities, type of prior antimicrobial treatment, preceding antimicrobial treatment by class (e.g., macrolides, tetracyclines, and fluoroquinolones), and detection of macrolide resistance gene mutations (20). To evaluate the factors associated with negative nasopharyngeal RT-PCR results among cases with positive oropharyngeal RT-PCR results, logistic regression analysis was also performed. Stata release 18 (StataCorp, College Station, TX, USA) was used for the statistical analyses.

## RESULTS

During the study period, 422 participants were subjected to simultaneous PCR testing using nasopharyngeal swabs for multiplex PCR and oropharyngeal swabs for the Smart Gene Myco test, of which 139 samples (32.9%) from nasopharyngeal swabs and 176 samples (41.7%) from oropharyngeal samples were PCR-positive for MP. Of the 176 positive samples, macrolide-resistance gene mutations were detected in 104 (59.1%) samples. A total of 133 cases tested positive for both multiplex PCR from nasopharyngeal samples and Smart Gene Myco from oropharyngeal samples, with six cases being only positive in nasopharyngeal swabs and 43 cases positive only in oropharyngeal swabs (Table 1). Of the 182 samples with positive PCR results either by the multiplex PCR or Smart Gene Myco test, the median age was 8.2 (interquartile range 5.3–10.9) years, and 68 (37.4%) were females (Table S1).

**TABLE 1:** Correlation table between multiplex PCR tests using nasopharyngeal swabs and Smart Gene Myco tests using oropharyngeal swabs^*a*^

	Smart Gene Myco		Total
	＋	−	
Multiplex PCR			
＋	133	6	139
−	43	240	283
Total	176	246	422

^*a*^ Abbreviation: PCR, polymerase chain reaction. Positive percent agreement 75.6% (133/176). Negative percent agreement 97.6% (240/246). Overall percent agreement 88.4% (373/422).

RT-PCR tests were conducted on all 422 residual extracts from oropharyngeal swabs and residual UTM from nasopharyngeal swabs, of which 136 (32.2%) nasopharyngeal and 183 (43.4%) oropharyngeal residual samples were positive. Among the 422 paired RT-PCR tests, 135, 1, and 48 were positive for both nasopharyngeal and oropharyngeal residues, only for a nasopharyngeal residue, and only for oropharyngeal residues, respectively. Table 2 shows the relationship between the multiplex PCR test from nasopharyngeal samples and the RT-PCR test from residual UTM from nasopharyngeal samples and between the Smart Gene Myco from oropharyngeal samples and the RT-PCR test from residual extracts from oropharyngeal samples.

**TABLE 2:** Relationships between the multiplex PCR test and the RT-PCR test from nasopharyngeal samples and between the Smart Gene Myco and the RT-PCR test from oropharyngeal samples^*a*^

Nasopharyngeal samples	Multiplex PCR		Total
	＋	−	
RT-PCR			
+	129	7	136
−	10	276	286
Total	139	283	422

Oropharyngeal samples	Smart Gene Myco		Total
	＋	−	
RT-PCR												
+	176	7	183									
−	0	239	239									
Total	176	246	422									

^*a*^ Abbreviation: RT-PCR, reverse transcription polymerase chain reaction; UTM, universal transport medium. The multiplex PCR test vs the RT-PCR test from nasopharyngeal samples: positive percent agreement 94.9% (129/136); negative percent agreement 96.5% (276/283); overall percent agreement 96.0% (405/422). The Smart Gene Myco and the RT-PCR test from oropharyngeal samples: positive percent agreement 96.2% (176/183); negative percent agreement 100% (239/239); overall percent agreement 98.3% (415/422).

With the RT-PCR test of the residual extract from oropharyngeal swabs as a reference, the sensitivity and specificity of detecting MP were 74.9% (95% CI 67.9%–81.0%) and 99.2% (97.0%–99.9%) in the multiplex PCR test from a nasopharyngeal swab, 96.2% (92.3%–98.4%) and 100.0% (98.5%–100.0%) in the Smart Gene Myco from an oropharyngeal sample, and 73.8% (66.8%–80.0%) and 99.6% (97.7%–100.0%) in the RT-PCR test using residual UTM from a nasopharyngeal sample, respectively. Compared with the sensitivity in the Smart Gene Myco using an oropharyngeal sample, those of the multiplex PCR test using the nasopharyngeal swab (*P* < 0.001) and RT-PCR test using residual UTM from a nasopharyngeal sample (*P* < 0.001) were inferior in the McNemar’s tests.

**Fig 1:**
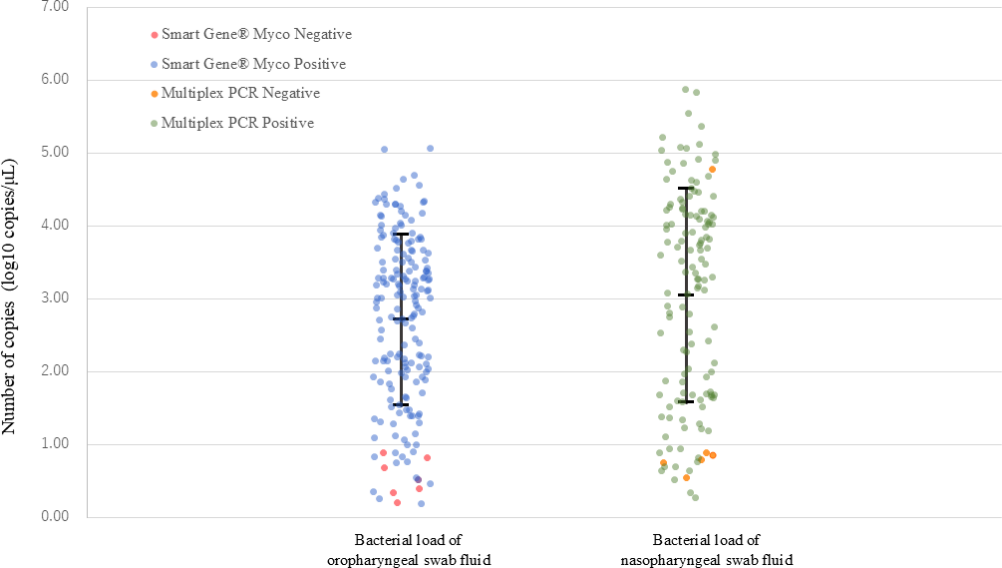
Nasopharyngeal and oropharyngeal DNA loads of *Mycoplasma pneumoniae* by qualitative results of commercially available PCR tests (a multiplex PCR respiratory panel and Smart Gene Myco). Error bars indicate geometric mean ± standard deviation.

**Fig 2:**
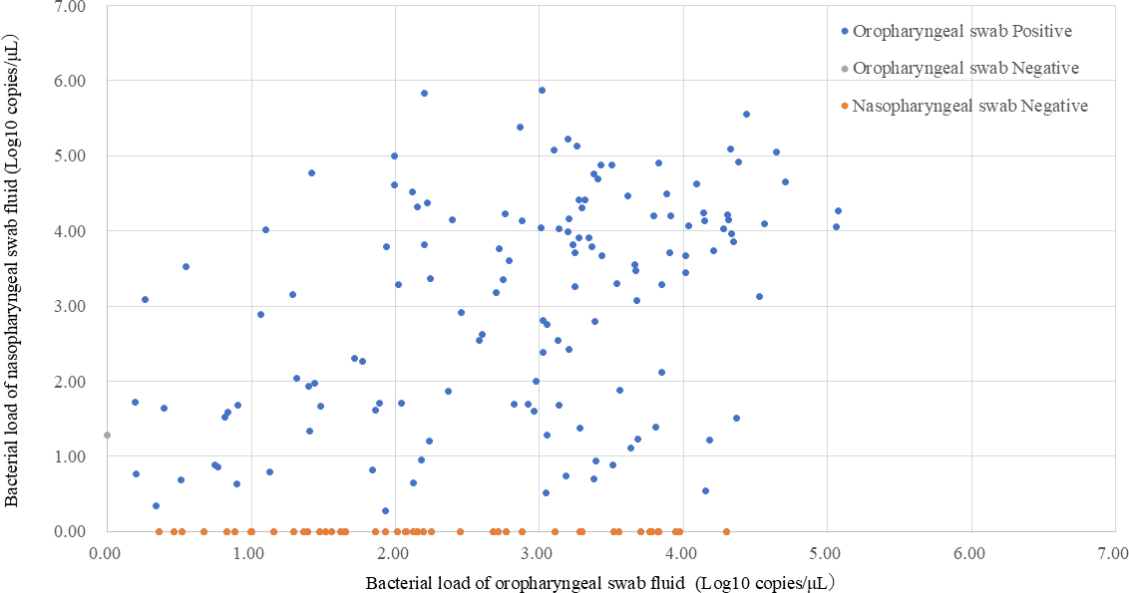
Correlation between nasopharyngeal and oropharyngeal DNA loads of *Mycoplasma pneumoniae.*

The DNA loads of nasopharyngeal and oropharyngeal swabs are shown in Fig. 1 and 2, respectively. Among the 183 samples that tested positive for oropharyngeal residual samples, DNA loads were significantly lower in samples with negative paired nasopharyngeal RT-PCR results than in those with positive results (*P* = 0.003 in Wilcoxon rank sum test). The DNA loads of all seven samples with a negative result in the Smart Gene Myco and a positive result in RT-PCR from oropharyngeal swabs were below 10 copies/μL. Fifteen cases had negative nasopharyngeal RT-PCR results and oropharyngeal DNA loads > 10^3^ copies/μL. Among the 135 pairs of samples with positive RT-PCR results from both nasopharyngeal and oropharyngeal residues, the Wilcoxon signed-rank test did not show a statistical difference in DNA loads (*P* = 0.077), whereas the Spearman’s rank correlation test showed a significant correlation of DNA loads between nasopharyngeal and oropharyngeal residues (*P* < 0.001).

In Table S2, preceding macrolide use or tetracycline use was not associated with oropharyngeal DNA loads (macrolides: coefficient −0.05 [95% CI −0.95, 0.84], *P* = 0.910 and tetracyclines: coefficient −1.09 [95% CI −2.19, 0.01], *P* = 0.052, respectively). A significant negative correlation was observed between preceding fluoroquinolone use and oropharyngeal DNA loads (coefficient −2.34 [95% CI −3.93, −0.76]; *P* = 0.004). The detection of macrolide resistance gene mutations was not significantly associated with DNA load (coefficient 0.66 [95% CI −0.20, 1.51], *P* = 0.134). In Table S3, the preceding antimicrobial use by class was not associated with the nasopharyngeal DNA loads (macrolides: coefficient −0.56 [95% CI −1.94, 0.82], *P* = 0.426, tetracyclines: coefficient −0.34 [95% CI −2.01, 1.34], *P* = 0.693 and fluoroquinolones: coefficient −1.42 [95% CI −4.22, 1.38]; *P* = 0.319, respectively).

The results of the logistic regression model used to evaluate the factors associated with negative nasopharyngeal RT-PCR results among cases with positive oropharyngeal RT-PCR results are presented in Table S4. None of the investigated covariates, including age, sex, presence of comorbidities, duration between symptom onset and sample collection, preceding antimicrobial use, and detection of macrolide resistance gene mutations, were statistically associated with negative nasopharyngeal RT-PCR results.

## DISCUSSION

To the best of our knowledge, this study has the largest sample size to date evaluating the diagnostic performance of MP PCR detection devices using both nasopharyngeal and oropharyngeal samples. The sensitivity of the Smart Gene Myco test using oropharyngeal samples was superior to that of the multiplex PCR test or the singleplex RT-PCR test using nasopharyngeal samples. This indicates that nasopharyngeal samples were inferior to oropharyngeal samples for MP detection.

Gnarpe et al. showed that MP was detected in six cases of oropharyngeal swabs, compared with two cases of nasopharyngeal swabs (21). Fujio et al. reported that RT-PCR detected MP in 15 cases from oropharyngeal swabs, compared with eight cases from nasopharyngeal swabs (4). Reznikov et al. showed that MP was detected in 11/22 (50%) of oropharyngeal swabs and in 9/20 (45%) of nasopharyngeal aspirates (5). In a study by Leber et al. with 58 patients, 27 (46.6%) were positive from both nasopharyngeal and oropharyngeal swabs, 31 (53.4%) were positive from oropharyngeal swabs only, and none were positive from nasopharyngeal swabs only (6). Honda et al. reported that the PCR detection rate was the highest from oropharyngeal swabs in 28/98 (28.6%) cases, compared with sputum 17/20 (14.2%) or bronchoalveolar lavage fluids 23/107 (21.5%) (22, 23). Some other studies suggested that a single oropharyngeal specimen had a high detection rate of MP among patients with MP pneumonia (24, 25). Our study added the evidence by providing a diagnostic performance of MP detection between nasopharyngeal and oropharyngeal swabs with the largest sample size to date.

While the oropharyngeal and nasopharyngeal DNA loads were significantly correlated, negative nasopharyngeal RT-PCR was observed, even when the DNA loads of their paired oropharyngeal samples were relatively high (>10^3^ copies/μL), which represents the median value observed in our positive samples and aligns with clinically significant thresholds suggested in previous studies (26). This indicates that the difference between the nasopharyngeal and oropharyngeal DNA loads may only partially explain the difference in sensitivity between these samples. Our logistic regression analysis showed no significant association between the investigated covariates (i.e., age, sex, comorbidity, duration between symptom onset and sampling, detection of macrolide resistance gene mutation, and prior antimicrobial exposure) and negative nasopharyngeal RT-PCR results among cases with positive oropharyngeal RT-PCR results. This suggests that false-negative PCR results from a nasopharyngeal sample may occur irrespective of patient characteristics, macrolide resistance in MP, or previous antimicrobial exposure.

Previous studies demonstrate that nasopharyngeal swabs are superior to oropharyngeal swabs for detecting certain respiratory viruses, whereas adenoviruses may be better detected by oropharyngeal swabs (27, 28). These previous findings, together with our study results, highlight the importance of selecting the sample type (i.e., nasopharyngeal versus oropharyngeal swabs) depending on the target pathogens in patients with respiratory infections. Whether the superiority of oropharyngeal samples over a nasopharyngeal sample in detecting MP, as confirmed in our study, can be applied to other atypical respiratory bacteria (e.g., *Chlamydia* and *Legionella*) requires further investigation (29, 30).

In our evaluation of the factors associated with oropharyngeal DNA load, fluoroquinolone use was negatively associated with oropharyngeal DNA load, whereas no significant association was noted with macrolide use (Table S4). This could be attributed to the higher prevalence of macrolide-resistant MP in Japan than in North America and Europe (10). In addition, while macrolides and tetracyclines are bacteriostatic antimicrobials, fluoroquinolones are bactericidal agents, which could explain the lower oropharyngeal DNA loads in fluoroquinolone use (31).

Currently, most commercially available *in vitro* diagnostics assays for respiratory pathogens are approved only for nasopharyngeal swabs, and the use of oropharyngeal swabs is not included in the package insert. Our findings indicate that oropharyngeal swabs may offer superior sensitivity for MP detection compared to nasopharyngeal swabs. Future efforts should focus on validating oropharyngeal swabs in larger, multi-center studies and seeking regulatory approval to broaden the clinical utility of these assays.

This study had some limitations. First, we could not evaluate whether the sample collection skills differed for each physician. Although resident physicians with less experience in obtaining pediatric samples may not have been as skilled as board-certified pediatricians, the same physician collected both oropharyngeal and nasopharyngeal samples from the same individual. While we could not evaluate human RNase *P* or beta-2 microglobulin, the measurement of these markers could have supported the evaluation of the presence of human mucosal cells in samples (32, 33). Another potential limitation was the inability to store some samples in the clinic at ultra-low temperatures (7% of all study samples for both nasopharyngeal and oropharyngeal swabs), although all paired samples were stored in the same condition. Furthermore, the Smart Gene Myco system used in this study only detects macrolide-resistance gene mutations at positions 2063 and 2064 in domain V of the 23S ribosomal RNA gene but does not detect other mutation sites such as C2617G, which is the third most common site for mutagenesis associated with macrolide resistance. This may need to be considered when interpreting our findings, although the nationwide surveillance showed that more than 95% of reported macrolide-resistance gene mutations in Japan were at positions of either 2063 or 2064 in domain V (34). Another important limitation of our study is the use of the RT-PCR test from oropharyngeal samples as the reference standard without adequate adjudication between different sample types. The differential detection observed between nasopharyngeal and oropharyngeal samples could potentially reflect intrinsic differences in the limits of detection, target copy number, or sensitivity of the assays when applied to different anatomical sites rather than true differences in bacterial presence. Without a robust adjudication process using multiple reference methods or culture-based confirmation, the apparent superior sensitivity of oropharyngeal sampling must be interpreted with caution. Future studies should address this limitation by incorporating additional reference standards or by validating RT-PCR performance characteristics specifically for each anatomical sampling site.

In conclusion, our study demonstrates that the sensitivity of MP detection is significantly higher in oropharyngeal samples than in nasopharyngeal samples. This study indicates that oropharyngeal samples should be used to detect MP rather than a nasopharyngeal sample. The results contribute to a potential change in the current recommendation regarding sampling methods to detect MP, according to national and international guidelines. Accurate identification of respiratory pathogens, including MP, is crucial not only for timely and appropriate antimicrobial treatment but also for efficient epidemiological surveillance.

### Key points

The PCR sensitivity of *Mycoplasma pneumoniae* (MP) was significantly better in oropharyngeal samples than in nasopharyngeal samples. Oropharyngeal samples should be used to detect MP rather than nasopharyngeal samples.

## Data Availability

Data supporting the findings of this study are available in the manuscript or its supplemental file. Additional data analysis may be available upon reasonable request to the corresponding author. The individual patient data are not publicly available because they contain information that can compromise the privacy of the study participants.

## SUPPLEMENTAL MATERIAL

Supplemental tables jcm.00458-25-s0001.docx Tables S1 to S4.

ASM does not own the copyrights to Supplemental Material that may be linked to, or accessed through, an article. The authors have granted ASM a non-exclusive, world-wide license to publish the Supplemental Material files. Please contact the corresponding author directly for reuse.
