# Supplementary material for: PCR sensitivity for Mycoplasma pneumoniae detection in nasopharyngeal and oropharyngeal swabs: a comparative study
Source: J Clin Microbiol. 2025 Jul 3;63(8):e00458-25. doi: 10.1128/jcm.00458-25 (PMC12345179; doi:10.1128/jcm.00458-25)
Supplement: Supplemental tables — Tables S1 to S4. [file jcm.00458-25-s0001.docx]

**Supplemental Table 1. Characteristics and co-detected respiratory viruses by nasopharyngeal multiplex PCR test among 182 participants with MP detection by a commercially available PCR test.**

|  | N=183 |
| --- | --- |
| Age (years) | Median 8.2 [IQR 5.3–10.9] |
| Female | 68 (37.4%) |
| Any respiratory virus detected | 85 (46.4%) |
| Adenovirus | 10 (5.5%) |
| Human metapneumovirus | 5 (2.7%) |
| Human rhinovirus/enterovirus | 78 (42.6%) |
| Influenza virus | 2 (1.1%) |
| Parainfluenza virus | 14 (7.7%) |
| Respiratory syncytial virus | 9 (4.9%) |
| Seasonal coronavirus | 0 (0.0%) |
| SARS-CoV-2 | 7 (3.8%) |
| Detection of macrolide-resistance gene mutation | 104 (59.1% ^a^) |
| Preceding macrolides | 61 (33.3%) |
| Preceding tetracyclines | 27 (14.8%) |
| Preceding fluoroquinolones | 11 (6.0%) |

Abbreviations: IQR; interquartile range, MP; *Mycoplasma pneumoniae*, RT-PCR; reverse transcription polymerase chain reaction, SARS-CoV-2; severe acute respiratory syndrome coronavirus 2

^a^ Of the 176 participants with positive SG Myco test from oropharyngeal swabs

**Supplemental Table 2. Generalized linear model for associations with oropharyngeal DNA load.**

|  | Coef | 95% CI low | 95% CI high | p |
| --- | --- | --- | --- | --- |
| Age (reference 12–17 years) |  |  |  |  |
| <6 years | 0.61 | -0.57 | 1.78 | 0.312 |
| 6–11 years | 0.19 | -0.87 | 1.25 | 0.726 |
| Sex (Female) | 0.08 | -0.71 | 0.88 | 0.835 |
| Comorbidity | -0.70 | -1.55 | 0.14 | 0.101 |
| Duration between onset to sampling | 0.05 | -0.04 | 0.13 | 0.310 |
| Preceding macrolides | -0.05 | -0.95 | 0.84 | 0.910 |
| Preceding tetracyclines | -1.09 | -2.19 | 0.01 | 0.052 |
| Preceding fluoroquinolones | -2.34 | -3.93 | -0.76 | 0.004 |
| Detection of MR gene mutation | 0.66 | -0.20 | 1.51 | 0.134 |

Abbreviations: CI, confidence interval; Coef, coefficient; MR, macrolide resistance; p, p-value

Generalized linear model with gamma distribution and identity link was conducted for log of oropharyngeal DNA loads.

**Supplemental Table 3. Generalized linear model for associations with nasopharyngeal DNA load.**

|  | Coef | 95% CI low | 95% CI high | p |
| --- | --- | --- | --- | --- |
| Age (reference 12–17 years) |  |  |  |  |
| <6 years | 1.62 | -0.20 | 3.43 | 0.082 |
| 6–11 years | 1.23 | -0.44 | 2.90 | 0.149 |
| Sex (Female) | -0.27 | -1.48 | 0.93 | 0.656 |
| Comorbidity | -1.32 | -2.58 | -0.05 | 0.041 |
| Duration between onset to sampling | 0.09 | -0.05 | 0.22 | 0.218 |
| Preceding macrolides | -0.56 | -1.94 | 0.82 | 0.426 |
| Preceding tetracyclines | -0.34 | -2.01 | 1.34 | 0.693 |
| Preceding fluoroquinolones | -1.42 | -4.22 | 1.38 | 0.319 |
| Detection of MR gene mutation | -0.21 | -1.55 | 1.14 | 0.762 |

Abbreviations: CI, confidence interval; Coef, coefficient; MR, macrolide resistance; p, p-value

Generalized linear model with gamma distribution and identity link was conducted for log of nasopharyngeal DNA loads.

**Supplemental Table 4. Logistic regression model for associations with negative nasopharyngeal RT-PCR among cases with positive oropharyngeal RT-PCR.**

|  | aOR | 95% CI low | 95% CI high | p |
| --- | --- | --- | --- | --- |
| Age (reference 12–17 years) |  |  |  |  |
| <6 years | 0.57 | 0.19 | 1.71 | 0.313 |
| 6–11 years | 0.73 | 0.28 | 1.88 | 0.515 |
| Sex (Female) | 0.53 | 0.24 | 1.14 | 0.105 |
| Comorbidity | 0.67 | 0.30 | 1.51 | 0.334 |
| Duration between onset to sampling | 0.98 | 0.90 | 1.06 | 0.620 |
| Preceding macrolides | 1.55 | 0.71 | 3.39 | 0.268 |
| Preceding tetracyclines | 0.49 | 0.21 | 1.16 | 0.106 |
| Preceding fluoroquinolones | 0.82 | 0.29 | 2.37 | 0.720 |
| Detection of MR gene mutation | 3.15 | 0.83 | 11.98 | 0.092 |

Abbreviations: aOR, adjusted odds ratio; CI, confidence interval; MR, macrolide resistance; p, p-value
